# Supplementary material for: Phase 1 Study to Evaluate the Safety of Reducing the Prophylactic Dose of Dexamethasone around Docetaxel Infusion in Patients with Prostate and Breast Cancer
Source: Cancers (Basel). 2023 Mar 9;15(6):1691. doi: 10.3390/cancers15061691 (PMC10046524; doi:10.3390/cancers15061691)
Supplement: Supplementary file 1 [file cancers-15-01691-s001.zip › cancers-2148862-supplementary.pdf]

Supplementary materials: Phase 1 Study to Evaluate the Safety of Reducing the Prophylactic Dose of Dexamethasone around Docetaxel Infusion in Patients with Prostate and Breast Cancer

Rieneke T. Lugtenberg [1,*,†], Stefanie de Groot [1,†], Danny Houtsma [2], Vincent O. Dezentjé [3,4], Annelie J. E. Vulink [3], Maarten J. Fischer [5], Johanneke E. A. Portielje [1,2], Jacobus J.M. van der Hoeven [1], Hans Gelderblom [1], Hanno Pijl [6] and Judith R. Kroep [1]

Table S1. Differences between dose levels for Glucose, Insulin and IGF-1 levels in patient with prostate cancer.

Dose level	1 (n=5)	2 (n=6)	3A (n=6)	3B (n=5)	p-value**
Glucose mmol/L*	6.92 (2.77)	6.57 (0.76)	7.01 (1.99)	5.87 (1.84)	0.77
Insulin mIU/L*	36.60 (39.39)	32.23 (23.64)	47.42 (72.56)	27.13 (33.23)	0.67
IGF-1 nmol/L*	16.10 (6.94)	16.36 (8.89)	16.86 (46.54)	18.03 (11.47)	0.92

* Median (IQR), **Kruskal Wallis test; Abbreviations: IGF-1: Insulin-like growth factor.

Table S2. Quality of Life scores for evaluable patients at baseline (Mean, SD).

**Prostate cancer**	**Cohort 1 (n=6)**	**Cohort 2 (n=6)**	**Cohort 3 (n=12)**	***p* value**
EORTC QoL-C30 domains				
Global Health	65.3 (33.9)	50.0 (26.4)	81.3 (17.9)	0.15
Physical functioning	73.3 (29.5)	80.0 (14.6)	92.8 (12.2)	0.20
Role functioning	63.9 (40.0)	72.2 (34.4)	88.9 (20.5)	0.34
Emotional functioning	80.6 (12.5)	84.7 (17.8)	84.0 (14.4)	0.44
Cognitive functioning	72.2 (37.5)	80.6 (16.4)	88.9 (16.4)	0.38
Social functioning	66.7 (42.2)	83.3 (21.1)	90.3 (20.7)	0.54
EORTC QoL-C30 symptoms				
Fatigue	38.9 (37.0)	27.8 (26.1)	13.0 (17.6)	0.62
Nausea	5.6 (8.6)	0.0 (0.0)	0.0 (0.0)	**0.04**
Pain	25.0 (39.1)	25.0 (25.3)	5.6 (19.2)	0.08
Dyspnea	11.1 (17.2)	27.8 (25.1)	13.9 (30.0)	0.35
Insomnia	33.3 (42.2)	22.2 (27.2)	16.7 (30.2)	0.73
Appetite loss	22.2 (40.4)	0.0 (0.0)	5.6 (13.0)	0.36
Constipation	5.6 (13.6)	11.1 (17.2)	2.8 (9.6)	0.41
Diarrhea	16.7 (40.8)	5.6 (13.6)	8.3 (15.1)	0.34
Financial difficulties	22.2 (40.4)	5.6 (13.6)	5.6 (13.0)	0.53
**Breast cancer**	**Cohort 1 (n=3)**	**Cohort 2 (n=6)**	**Cohort 3 (n=1)**	***p* value**
EORTC QoL-C30 domains				
Global Health	66.7 (16.7)	73.6 (26.0)	66.7	0.35
Physical functioning	77.8 (20.4)	86.7 (8.2)	80.0	0.40
Role functioning	55.6 (9.6)	58.3 (32.9)	33.3	0.12
Emotional functioning	91.7 (8.3)	77.8 (21.5)	66.7	0.08

Cognitive functioning	77.8 (9.6)	75.0 (17.5)	66.7	0.77
Social functioning	88.9 (19.2)	69.4 (38.6)	66.7	0.40
EORTC QoL-C30 symptoms				
Fatigue	37.0 (17.0)	50.0 (33.5)	55.6	0.82
Nausea	11.1 (9.6)	20.0 (21.7)	0.0	0.68
Pain	27.8 (9.6)	25.0 (37.6)	0.0	0.21
Dyspnea	11.1 (19.2)	38.9 (25.1)	33.3	0.41
Insomnia	55.6 (19.2)	33.3 (36.5)	33.3	0.29
Appetite loss	0.0 (0.0)	33.3 (21.1)	66.7	**0.03**
Constipation	55.6 (50.9)	22.2 (27.2)	0.0	0.57
Diarrhea	11.1 (19.2)	11.1 (17.2)	33.3	0.44
Financial difficulties	22.2 (19.2)	5.6 (13.6)	0.0	0.24

Abbreviations: EORTC: European Organization for Research and Treatment of Cancer-Core, QoL: Quality of Life.

Table S3. Changes in scores from baseline for patients with prostate cancer.

		**Questionnaire time points**			**p-value**		
		**Baseline**	**After 3 cycles**	**After 6 cycles**	**Time**	**Cohort**	**Time by Cohort**
Global Health	Cohort 1	68.3	86.1	70.0	0.84	0.05	0.63
	Cohort 2	36.11	45.8	54.2			
	Cohort 3	78.3	77.1	72.9			
**Functioning scales**							
Physical functioning	Cohort 1	77.3	93.3	81.3	**0.03**	0.11	**0.03**
	Cohort 2	71.1	56.7	60.0			
	Cohort 3	91.3	90.8	74.1			
Role functioning	Cohort 1	70.0	77.8	66.7	0.06	0.12	0.06
	Cohort 2	44.4	16.7	33.3			
	Cohort 3	86.7	79.2	60.4			
Emotional functioning	Cohort 1	83.3	83.3	81.7	0.77	0.31	0.81
	Cohort 2	72.2	70.8	58.3			
	Cohort 3	83.3	85.4	82.3			
Cognitive Functioning	Cohort 1	66.7	88.9	73.3	0.44	0.34	0.44
	Cohort 2	72.2	75.0	91.7			
	Cohort 3	86.7	91.7	83.3			
Social functioning	Cohort 1	73.3	88.9	73.3	0.49	0.34	0.59
	Cohort 2	77.8	50.0	58.3			
	Cohort 3	91.7	89.6	83.3			
**Symptoms**							
Fatigue	Cohort 1	35.6	40.7	48.9	**0.02**	0.13	0.06
	Cohort 2	48.1	61.1	66.7			
	Cohort 3	14.4	23.8	37.5			
Nausea	Cohort 1	6.7	0.0	6.7	**0.03**	0.20	**0.04**
	Cohort 2	0.0	0.0	16.7			
	Cohort 3	0.0	0.0	6.3			
Pain	Cohort 1	30.0	5.6	3.3	0.53	0.23	0.06
	Cohort 2	38.9	33.3	50.0			

	Cohort 3	6.7	12.5	35.4			
Dyspnea	Cohort 1	13.3	0.0	33.3			
	Cohort 2	33.3	16.7	50.0			
	Cohort 3	13.3	8.3	33.3	**<0.01**	0.39	**0.02**
Insomnia	Cohort 1	26.7	33.3	33.3			
	Cohort 2	33.3	16.7	66.7			
	Cohort 3	20.0	8.3	33.3	0.18	0.60	0.40
Appetite loss	Cohort 1	26.7	0.0	20.0			
	Cohort 2	0.0	0.0	50.0			
	Cohort 3	6.7	0.0	12.5	0.69	0.16	**0.04**
Constipation	Cohort 1	6.7	0.0	6.7			
	Cohort 2	22.2	0.0	16.7			
	Cohort 3	3.33	4.2	4.2	0.56	0.17	0.93
Diarrhea	Cohort 1	20.0	11.1	20.0			
	Cohort 2	0.0	0.0	0.0			
	Cohort 3	10.0	4.2	12.5	0.76	0.45	0.98
Financial difficulties	Cohort 1	26.7	0.0	13.3			
	Cohort 2	11.1	0.0	0.0			
	Cohort 3	3.33	4.2	0.0	0.08	0.10	**0.02**

P Time: changes of QoL scores over time; P Cohort: differences in QoL scores between cohorts; P Time by Cohort: different effects between cohorts over time.

Table S4. (A)Occurrence of hypersensitivity reactions (HSR) and fluid retention in evaluable patients with breast cancer; (B) Occurrence of hypersensitivity reactions (HSR) and fluid retention in evaluable patients with prostate cancer.

(A) Occurrence of hypersensitivity reactions (HSR) and fluid retention in evaluable patients with breast cancer

Patient	*Cohort*	*TNM classification*	*Receptor status*	*Treatment*	*Docetaxel treatment*	*Cycles of Docetaxel*	*Cumulative dose of Docetaxel*	*HSR (any grade)*	*HSR (grade III/IV)*	*Fluid retention (any grade)*	*Fluid retention (grade III/IV)*
1102	1	T2N0M0	TNBC	Neo-adjuvant	Monotherapy 100 mg/m²	4	375 mg/m²	-	-	-	-
1104	1	T1N0M0	ER+, PR/Her2-	Adjuvant	Monotherapy 100 mg/m²	4	400 mg/m²	-	-	-	-
1105	1	T2N1M0	ER/PR+, Her2-	Adjuvant	Monotherapy 100 mg/m²	4	340 mg/m²	-	-	-	-
1106	1	T2N1M0	ER/PR+, Her2-	Neo-adjuvant	Monotherapy 100 mg/m²	4	340 mg/m²	-	-	-	-
1107	1	T1N0M0	ER/PR+, Her2-	Adjuvant	Monotherapy 100 mg/m²	4	325 mg/m²	-	-	-	-
1108	1	T1N1M0	ER/PR+, Her2-	Adjuvant	Monotherapy 100 mg/m²	4	325 mg/m²	-	-	Grade 1 edema	-
1201	2	T2N1M0	ER/PR+, Her2-	Neo-adjuvant	Monotherapy 100 mg/m²	4	340 mg/m²	-	-	Grade 2 edema	-
1202	2	T3N3M0	TNBC	Neo-adjuvant	Monotherapy 100 mg/m²	4	307 mg/m²	-	-	-	-
1203	2	T2N0M0	TNBC	Neo-adjuvant	Monotherapy 100 mg/m²	4	400 mg/m²	-	-	-	-
1204	2	T1N0M)	ER/PR+, Her2-	Adjuvant	Combination therapy* 75 mg/m²	4	300 mg/m²	-	-	-	-
1205	2	T2N1M0	ER+, PR/Her2-	Neo-adjuvant	Monotherapy 100 mg/m²	1	100 mg/m²	-	-	-	-
1206	2	T2N0M0	TNBC	Neo-adjuvant	Monotherapy 100 mg/m²	4	400 mg/m²	-	-	-	-
1303	3	T1N1M0	ER+, PR/Her2-	Neo-adjuvant	Monotherapy 100 mg/m²	6	600 mg/m²	-	-	-	-

1304	3	T2N1M1	ER/PR+, Her2+	Palliative	Combination therapy[**] 100 mg/m²	6	450 mg/m²	-	-	-	-

(B) Occurrence of hypersensitivity reactions (HSR) and fluid retention in evaluable patients with prostate cancer

Patient	*Cohort*	*Disease*	*Treatment*	*Docetaxel treatment*	*Cycles of Docetaxel*	*Cumulative dose of Docetaxel*	*HSR (any grade)*	*HSR (grade III/IV)*	*Fluid retention (any grade)*	*Fluid retention (grade III/IV)*
2101	1	mCRPC	Palliative	Monotherapy 75 mg/m²	1	75 mg/m²	-	-	-	-
2102	1	mCRPC	Palliative	Monotherapy 75 mg/m²	9	645 mg/m²	-	-	-	-
2103	1	mHSPC	Palliative	Monotherapy 75 mg/m²	6	450 mg/m²	-	-	-	-
2104	1	mHSPC	Palliative	Monotherapy 75 mg/m²	6	450 mg/m²	-	-	-	-
2105	1	mCRPC	Palliative	Monotherapy 75 mg/m²	8	600 mg/m²	-	-	-	-
2106	1	mHSPC	Palliative	Monotherapy 75 mg/m²	6	450 mg/m²	-	-	Grade 2 edema	-
2201	2	mCRPC	Palliative	Monotherapy 75 mg/m²	6	450 mg/m²	-	-	-	-
2202	2	mCRPC	Palliative	Monotherapy 75 mg/m²	5	375 mg/m²	-	-	-	-
2203	2	mCRPC	Palliative	Monotherapy 75 mg/m²	6	450 mg/m²	-	-	-	-
2204	2	mCRPC	Palliative	Monotherapy 75 mg/m²	3	225 mg/m²	-	-	-	-
2205	2	mCRPC	Palliative	Monotherapy 75 mg/m²	6	450 mg/m²	-	-	-	-
2206	2	mHSPC	Palliative	Monotherapy 75 mg/m²	6	450 mg/m²	Grade 1[**]	-	-	
2207	2	mHSPC	Palliative	Monotherapy 75 mg/m²	6	450 mg/m²				
2301	3A	mCRPC	Palliative	Monotherapy 75 mg/m²	5	281 mg/m²				
2302	3A	mHSPC	Palliative	Monotherapy 75 mg/m²	6	450 mg/m²				
2303	3A	mHSPC	Palliative	Monotherapy 75 mg/m²	6	450 mg/m²				
2304	3A	mHSPC	Palliative	Monother-	6	450 mg/m²	-	-	Grade 1	-

				apy 75 mg/m^2					edema	
2305	3A	mHSPC	Palliative	Monotherapy 75 mg/m^2	6	450 mg/m^2	-	-	-	-
2306	3A	mCRPC	Palliative	Monotherapy 75 mg/m^2	2	150 mg/m^2	-			
2401	3B	mHSPC	Palliative	Monotherapy 75 mg/m^2	6	450 mg/m^2				
2402	3B	mHSPC	Palliative	Monotherapy 75 mg/m^2	5	356 mg/m^2				
2403	3B	mHSPC	Palliative	Monotherapy 75 mg/m^2	6	450 mg/m^2	-	-	Grade 1 edema	-
2405	3B	mHSPC	Palliative	Monotherapy 75 mg/m^2	6	450 mg/m^2	-	-	Grade 2 edema	-
2407	3B	mHSPC	Palliative	Monotherapy 75 mg/m^2	6	450 mg/m^2	-	-	-	-
2409	3B	mHSPC	Palliative	Monotherapy 75 mg/m^2	6	450 mg/m^2	-	-	-	-

(A)* Docetaxel – cyclophosphamide ** Docetaxel – trastuzumab – pertuzumab; Abbreviations: TNBC: triple negative breast cancer; ER: Estrogen receptor; PR: progesterone receptor; Her2: human epidermal growth factor receptor 2; (B)** After a grade 1 HSR at the first cycle of docetaxel this patient decided to use the normal dosage of dexamethasone in the consequent cycles and he went off study; Abbreviations: mHSPC: metastatic Hormone-sensitive prostate cancer; mCRPC: metastatic castration-resistant prostate cancer.
